# Experimental Study on Interfacial Friction Characteristics of Reinforced Clay

**DOI:** 10.3390/polym14214626

**Published:** 2022-10-31

**Authors:** Chenyang Zhang, Hong Mei, Guochang Hu, Jin Liu, Jian Xue, Xiaoyong Zhu, Hongning Lu, Zezhuo Song, Wenyue Che

**Affiliations:** 1 School of Earth Sciences and Engineering, Hohai University, Nanjing 210098, China; 2 First Geological Brigade of Jiangsu Geology & Mineral Exploration Bureau, Jiangsu Geological Bureau, Nanjing 210000, China; 3 Jiangsu Institute of Geology and Minerals Investigation, Jiangsu Geological Bureau, Nanjing 210000, China

**Keywords:** clay, sisal fiber, polyvinyl acetate, sliding model test, reinforced mechanism

## Abstract

Clay is one of the important base materials in slope restoration. The adhesion of clay–rock interface plays a decisive role in the repairing effect on rock slopes. Fibers and polymers are widely used as a clay improvement method in rock slope repair. In this paper, the friction effect of sisal fiber and polyvinyl acetate (PVAc)-reinforced clay was studied through the design of an indoor rock-like interface sliding model test. Using modelled test results and scanning electron microscope (SEM) images, the reinforced clay was analyzed. The test results showed that the critical sliding angle and maximum static friction force of clay decreased with the increase of moisture content. An excess of fiber content and moisture content weakens the coupling effect of fiber-anchoring clay. Fiber content of 0.8% and PVAc content of 2% had the best effect on enhancing the sliding resistance of clay and provided good adhesion for dangerous interfaces of rock slope at 35° and 45°, respectively. PVAc formed a three-dimensional networked elastic membrane structure to improve the skid resistance and dynamic friction coefficient of the clay. The results provide an effective way for soil improvement and ecological restoration.

## 1. Introduction

With the rapid development of the economy, a large number of exposed rock slopes have been formed by engineering excavation and mining, which seriously damage the ecological environment and result in disasters such as debris flows, rockfalls and landslides [1,2]. At present, soil spraying, plant growth bag, fertilizer bag and other commonly-used slope protection methods could provide a growth environment for vegetation on high and steep rock slopes [3]. The principle of soil spraying is to improve the clay, which could be in situ or obtained from other places, by spraying it on the rock slope using a high-pressure sprayer. The double-layer structure of guest soil layer and rock layer is formed on the rock slope, which provides sufficient environmental conditions for plant growth. The ecological restoration of the rock slope can increase the strength, anti-erosion ability and water stability ability of the guest soil substrate using the complex structural characteristics of the rock mass structural plane and the anchoring effect of plant roots. However, the adhesion of surface soil that influences the relative displacement between the overlying soil and the underlying rock is usually poor after being washed by rainwater and weathered, which results in the loss of suitable conditions for vegetation growth on the slope surface. Therefore, how to strengthen the surface soil to maintain high mechanical coupling with the rock interface is of great significance to solving the surface stability of the rock slope. 

At present, the frequently-used slope reinforcement methods, such as anti-slide piles, retaining walls, concrete grouting and so on, can meet the stability requirements of slopes in the process of slope protection [4,5,6]. However, these methods rarely act on the soil surface, and fail to solve the problem of poor coupling between the soil and rock interface and meet ecological requirements. Clay improvement technology is currently widely used to improve the engineering properties of clay to meet the requirements of slope stability, which is mainly divided into physical reinforcement methods and chemical additives [7,8,9,10]. As a new eco-physical reinforcement method known as fiber reinforcement has become increasingly mature. Compared with the traditional reinforcement method, it has the following advantages: (i) fiber has excellent strength and elastic characteristics [11,12]; (ii) easy construction and easy mixing with soil. Based on these advantages, fiber-reinforced clay has attracted more and more attention from international scholars and achieved some remarkable results [13]. Different types of fibers have different properties. For synthetic fibers (i.e., glass fiber, polypropylene fiber and polyester), they have good strength and elastic characteristics [14]. For natural fibers (i.e., sisal fiber, palm fiber and lignin fiber), their strength and elastic characteristics are smaller than those of synthetic fibers. However, their strength and elastic characteristics could meet the needs of engineering. In addition, natural fibers have good environmental friendliness [15]. Under the influence of water, natural fibers can degenerate. But this is a long process, which would not affect the effect of fiber on improving clay [16]. Traditional chemical reinforcement methods include inorganic material additives such as cement and lime, which improve the pH and brittleness of the clay after reinforcement. As a new type of reinforcement material, polymer curing agent has advantages of high economic benefit and environmental protection [17,18,19]. In addition, a large number of achievements have been made in polymer clay stabilizers [20,21,22].

In traditional clay test research, researchers tend to study the strength properties of clay itself, such as shear strength and compressive strength. In actual geological structure and engineering, the interfacial friction involving the clay–rock interface is often ignored. Zhang [23,24] and He [25] carried out cyclic loading tests and direct shear tests on large-scale contact surfaces and concluded that contact surface roughness increased friction angle and shear strength. Therefore, the interfacial friction property of the clay–rock interface is also an important research object and has gradually attracted attention [26]. Li [27] pointed out that accumulation landslides mainly slide along the bedrock’s surface, and its shape has a great influence on the slip and revival of the accumulation. Zhang [28] studied the starting conditions and sliding mechanisms induced by rainfall through a physical model test and pointed out that slope was the potential factor inducing interface landslide. Due to the unicity and unrepeatability of sliding failure parameters caused by the interface of overlying clay-rock slope in the field, it is impossible to separate different influencing factors for single-factor study in practical research. As a result, the data obtained from the field survey cannot accurately reflect the influence of various factors on the movement characteristics of clay.

Based on the above, this paper combines the advantages of the laboratory test method to carry out the interface sliding test of different properties of the overlying clay layer using the laboratory model test. To modify the poor conditions of the overlying clay layer, fiber reinforcement technology and polymer-reinforced technology were used as improvement methods to simulate the sliding change rule of the overlying clay layer under different fiber reinforcement ratios and curing agent contents. Through studying the clay-rock contact slip mechanism and possible failure modes before and after improvement, this test provides a theoretical basis for the prevention and control of sliding failure in clay–rock binary structures and the design of ecological slope protections, which have prominent theoretical and practical significance [29].

## 2. Materials and Methods

### 2.1. Test Device

**Figure 1:**
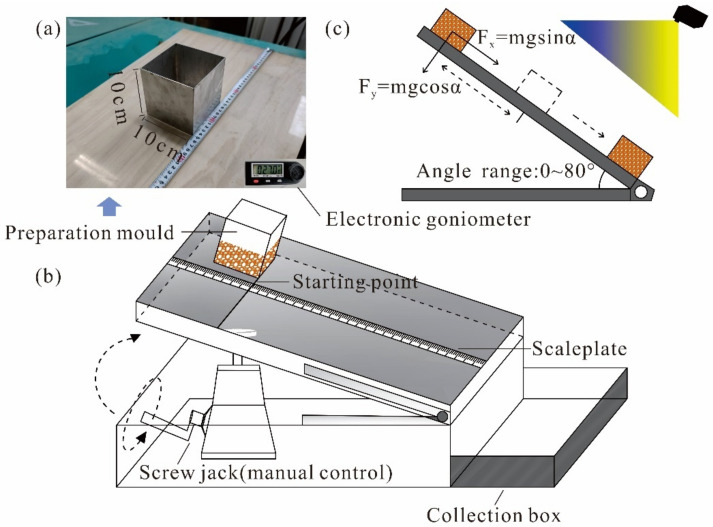
Model test devices: (**a**) sample preparation mold; (**b**) indoor sliding test model; (**c**) angle range and force analysis.

The indoor model test device, which was developed for this study, consists of a sample preparation mold, angle adjusting device, collection device and digital camera. The sample preparation mold is a customized aluminum square box without top and bottom surfaces (Figure 1a). The angle adjustment device is composed of a tilting plate, ruler, automatic electronic angle-measuring instrument, and lifting device (Figure 1b). The lifting equipment is composed of an improved lengthened spiral jack. During the test, the tilt plate is lifted (3°/s) by rotating the rocker manually, and the inclination angle is adjusted between 0 and 80°. The angle measured in the test is finally recorded automatically by the electronic goniometer. A camera (60 fps) is responsible for recording the whole process of the sample displacement (Figure 1c).

### 2.2. Sisal Fiber

**Figure 2:**
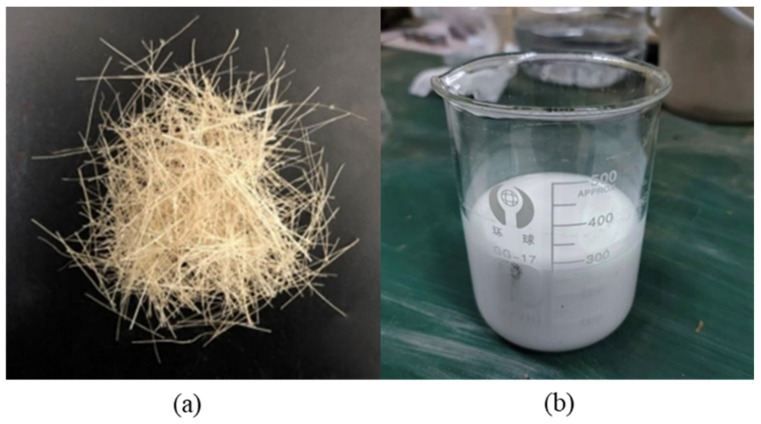
Reinforced materials: (**a**) 2 cm sisal fiber; (**b**) polyvinyl acetate.

The fiber material used in the experiment is sisal fiber (Figure 2a). Sisal fiber belongs to medium strength and high elastic fiber, which is a natural fiber widely used at present [30]. Sisal fiber has the characteristics of friction resistance, corrosion resistance, salt and alkali resistance, high elasticity, etc. Its basic physical parameters are shown in Table 1, which were provided by manufacturer Guangxi Sisal Group Co., Ltd., Nanning, China.

### 2.3. Polyvinyl Acetate Polymer Stabilizer

In this experiment, PVAc was used as a contrast chemical test for fiber improvement. Its basic physical parameters are shown in Table 2, which were provided by manufacturer Changzhou Wanhongxin Material Co., Ltd., Changzhou, China. PVAc (Figure 2b) is a new type of ecological polymer clay stabilizer which is diluted into white liquid as a way of test preparation. The main components of PVAc include vinyl acetate (VA) and ammonium persulfate (APS). There are many long-chain macromolecules and polar carboxyl groups (–OOCCH_3_) at the end of the prepolymer, which was synthesized using persulfate as initiator in the polymerization reaction. PVAc can be effectively used for clay reinforcement and ecological slope restoration. The advantages of PVAc include: (i) easy to produce; (ii) has high applicability and good economic benefits; (iii) can increase the stress strength of clay [31].

### 2.4. Test Methods and Processes

**Figure 3:**
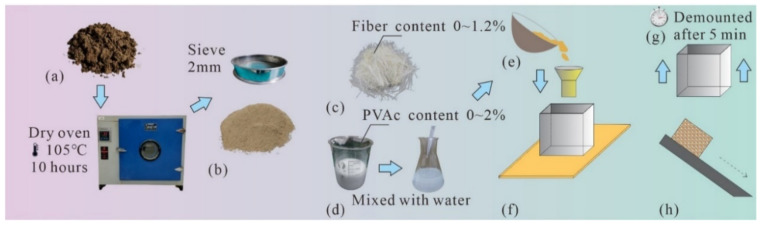
Model test materials and processes: (**a**) undisturbed clay; (**b**) clay that has been sifted after drying in 105 °C oven for 10 h; (**c**) 2 cm sisal fiber; (**d**) PVAc and its diluent; (**e**) specimen preparation; (**f**) custom-made mold; (**g**) curing time; (**h**) clay sample.

Before the start of the test, undisturbed clay (Figure 3a) from the Jiangning area of Nanjing was dried in an oven and mechanically crushed with 2 mm sieve (Figure 3b). Then the liquid–plastic limit and plastic index of the test clay samples were obtained through the limit moisture content test. The physical properties are shown in Table 3, which were tested in a laboratory according to Chinese National Standards GB/T 50123-2019. The detailed process of the laboratory model test emulsion is described in the following steps and presented in Figure 3e–h.

Step 1: The inner wall of the sample preparation mold was smeared with Vaseline and placed on the tilting plate, and a fixed scale was aligned as the initial height of the test.

Step 2: The quality of water, fiber, PVAc, and clay were weighed to blend according to the test scheme. The fiber content and PVAc content are shown in Table 4. Then the mixture was slowly poured into the mold box through the funnel at a certain height on the upper part of the mold to make it naturally accumulate due to force of gravity, so that the bottom surface of the sample could fully come into contact with the acrylic plate interface. The principle of stratified sample preparation was adopted in this test, and the thickness of each layer was approximately 1cm to avoid large pores in the clay sample.

Step 3: After completion of accumulation, the sample surface was smoothed and sealed standing for 5–15 min to facilitate the full mixing of reinforcement materials with clay. 

Step 4: Removed the mold from the top, then slowly lifted the tilting plate. When the sample changed from static state to sliding state, stopped lifting the tilting plate, and recorded the critical angle with a electronic inclinometer, which was recorded as the tilting angle α. By calculating the average value and standard deviation of three parallel samples, the influence of moisture content and fiber content/PVAc content could be analyzed by multi-factor analysis of variance (ANOVA).

Step 5: The entire process of clay specimen sliding was recorded with a camera. The camera automatically took photos and reserved at a time interval of 1 s. The distance of sample displacement per second was recorded by ruler and drawn into an image for further analysis.

After reading the photos automatically taken with a camera, the sliding distance *S* and time *t* of the sample were recorded and mapped into a scatter plot. The fitting curve *S*(*t*) of the sliding displacement with different parameters of the clay–rock interface were obtained by fitting the data with appropriate curve types. The second derivative of time *t* was calculated for the fitting function *S*(*t*), and the fitting acceleration of the specimen in the sliding displacement process of clay–rock interface can be chalked up as follows [32]:

*a* = *g* × (sin*α* − *μ* × cos*α*), (1)

By transforming Equation (1), we obtain

*μ* = tan*α* − *a*/(*g* × cos*α*). (2)

In this formula: *α* is the tilting angle; *g* is the gravity acceleration; the value of *g* is 9.8 m/s^2^; and *μ* is the dynamic friction coefficient of clay-rock interface (between sample and tilting plate).

## 3. Results and Discussion

### 3.1. Tilting Angle and Sliding Force Analysis

**Figure 4:**
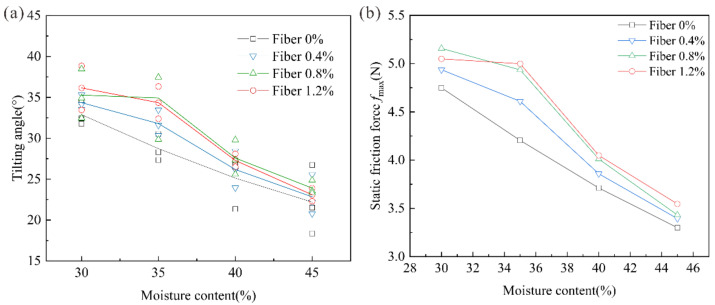
Model test result of sisal fiber-reinforced clay: (**a**) The tilting angle of clay sample; (**b**) Maximum static friction of clay sample.

Figure 4a shows the transformation law between the tilting angle and moisture content under three sets of fiber contents. It can be seen from Figure 4a that the tilting angle of pure clay was generally distributed from 20° to 34°, showing a decreasing trend with the increase of moisture content. The tilting angle of fiber-reinforced clay was distributed from 22.84 ± 2.46° to 38.84 ± 2.69°; however, the fiber-reinforced clay had a superior tilting angle value when the moisture contents were 30% and 35%. Combined with Table 5, the tilting angles of 0.8% fiber-reinforced clay were 38.84 ± 2.69° and 36.32 ± 1.97°, which increased by 18% and 26%, respectively, compared with pure clay. When the moisture contents were 40% and 45%, the corresponding critical tilting angle values were 28.09 ± 0.8° and 23.18 ± 0.8°, which increased by 12% and 4%, respectively. The tilting angle of fiber-reinforced clay decreased with the increase in moisture content, which indicated that the increase in moisture content weakened the strengthening effect of sisal fiber to some extent, resulting in a decrease in the coupling degree between clay particles and sisal fiber, has also been proven by the research results of other scholars. Zhang et al. [33,34] found that the interfacial friction characteristics of clay reduced with the increase in moisture content.

The fiber content also had a certain effect on the coupling degree between clay samples and sisal fiber. It can be seen from Figure 4a that the critical inclination angle of the clay sample generally increased slowly when the fiber content was 0.4%. When the fiber content increased to 0.8%, the critical inclination angle of the sample increased greatly at a moisture content of 30–40%. When the fiber content increased to 1.2%, the moisture content was 45% and the critical inclination angle of the sample increased slightly; however, the improvement effect of the sample was not as good as that of fiber content at 0.8%. 

By calculating the maximum static friction force (Figure 4b), it can be seen that the maximum static friction force of the samples after fiber reinforcement were significantly increased when the moisture content of the samples were 30% and 35%. The sample’s static friction force of 0.8% fiber content was higher than the sample when the fiber contents were 0.4% and 1.2%. When the moisture content was higher than 35%, the maximum static friction force of the fiber-reinforced samples were close to pure clay, and the effect of fiber reinforcement on the slip-resistance stability for improving clay was not obvious.

**Figure 5:**
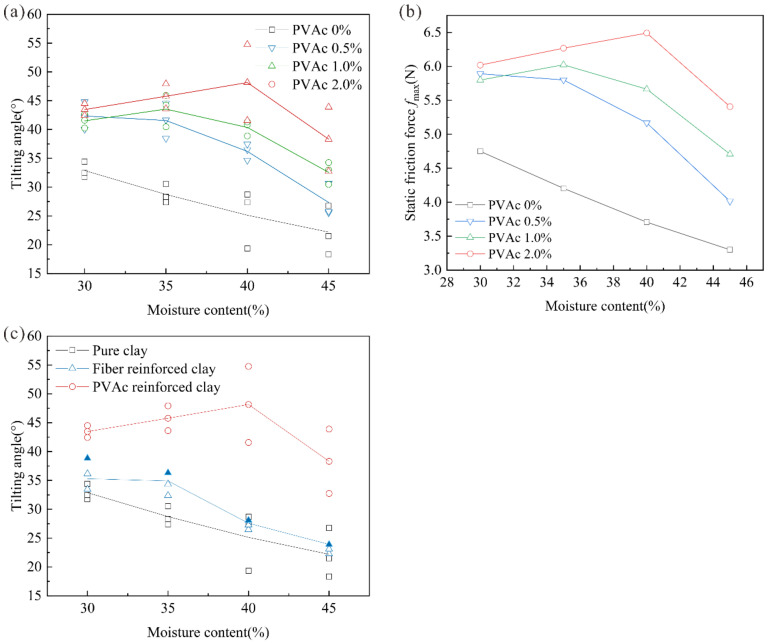
Model test result of PVAc-reinforced clay: (**a**) The tilting angle of clay sample; (**b**) Maximum static friction of clay sample; (**c**) The tilting angle range of differently reinforced clays.

Figure 5a shows the transformation law between the tilting angle and moisture content under three different PVAc contents. It can be seen from Figure 5a that the critical tilting angle of the reinforced clay with PVAc polymer curing agent was generally distributed between 25° and 45°, which had an obvious improvement effect compared with pure clay. The critical tilting angle of the sample increase was obvious with the increase in curing agent content. Combined with Table 5, the 2% PVAc content’s rangeability of the critical tilting angle was significantly higher than the samples with 0.5% and 1% PVAc content. Compared with pure clay, the critical tilting angles of the 2% PVAc sample under the four moisture content conditions were 43.47 ± 1.03°, 45.78 ± 2.15°, 48.16 ± 6.59°, and 38.32 ± 5.58°, which increased by 32%, 59%, 92%, and 72%, respectively, proving that the increase of PVAc content has a great effect on the clay improvement of the sliding condition. In addition, under the condition of high moisture content (40–45%), the critical tilting angle of the sample was lower than 30°, while the sample with 2% PVAc content still maintained a higher angle value. 

By calculating the maximum sliding static friction force, as shown in Figure 5b, it was obvious that the maximum sliding static friction force of clay was improved by PVAc. When the moisture content was higher than the liquid limit, the maximum static friction force of the 2% PVAc clay sample was higher than that of the 0.5% and 1% clay samples. When the PVAc content was low, it was difficult to assess the full effectiveness of the curing agent with the thin “gel conjunctiva” by mixing PVAc diluent with clay. When the content of PVAc was further increased, the cement particles on the surface of the clay formed a holistic cementation structure with high strength properties between PVAc solution and clay particles, so it had good resistance to sliding force and increased the maximum static friction force of the sample. 

As can be seen from the comparison of the critical angle between fiber and PVAc hardening agent (Figure 5c), PVAc-reinforced clay can meet the rock mass contact surface with the angle of more than 40°. This kind of contact surface often occurs in high and steep rock slopes, which is difficult and dangerous for ecological restoration. The fiber-reinforced clay can satisfy the ecological restoration of the contact surface of steep slope rock when the moisture content is low; however, the coupling effect of fiber-reinforced clay and rock contact surface is poor when the moisture content or contact surface angle is too high, and it is difficult to meet the intensity requirements of ecological restoration.

Multivariate analysis of variance (ANOVA) is used to study the difference relationship between two or more definite data X and one quantitative data Y, which is usually used in experimental studies. In this paper, multivariate analysis of variance was used to study the relationship between moisture content and fiber/PVAc content on the critical angle. From the analysis results, it can be seen that moisture content presented significantly (F = 60.344, *p* = 0.000 < 0.05), indicating that the main effect of moisture content existed, and moisture content had a significant impact on the critical angle. The results of fiber content were still significant (F = 4.613, *p* = 0.007 < 0.05), indicating that the main effect still existed and the influence of fiber content on critical angle could not be ignored. Similarly, it indicated that water content (F = 19.893, *p* = 0.000 < 0.05) and PVAc content (F = 45.491, *p* = 0.000 < 0.05) were significant to the test results. Therefore, it was necessary to further analyze the influence of water content and reinforcement material content on the angle results from the interface sliding test.

### 3.2. Sliding State Analysis

Landslide failure of the slope is instantaneous and unpredictable. Rainfall is one of the main factors leading to landslide [35]. In in situ field tests, it is difficult to obtain accurate data from a bad geographical environment. Therefore, the indoor model test is used to simulate the displacement behavior of the slope interface, which has guiding significance for solving the instability problem of slopes under high water content.

**Figure 6:**
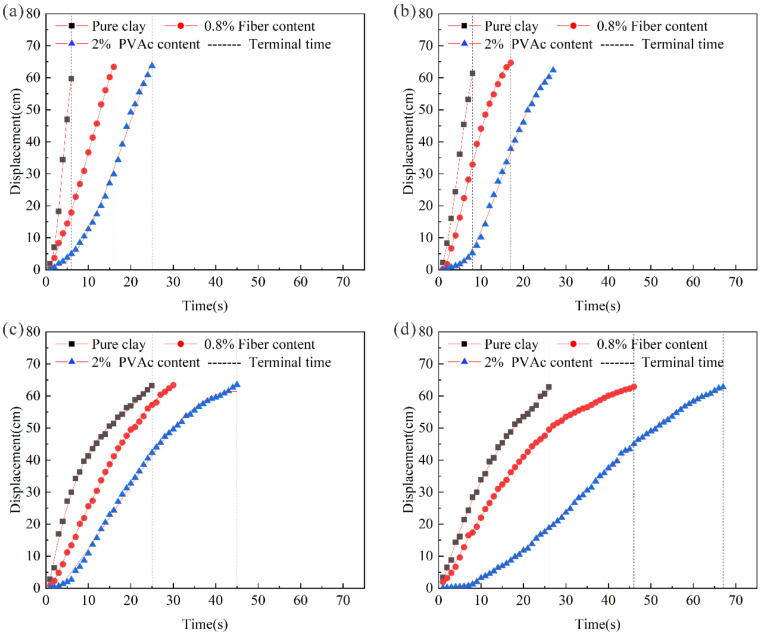
Time displacement curve at moisture contents of (**a**) 45%, (**b**) 40%, (**c**) 35%, and (**d**) 30%.

In order to study the reinforcement influence of fiber and PVAc on the sliding process of the sample, a time-displacement equation of specimen sliding was calculated based on video image analysis and curve fitting methods. It turns out that there is a significant nonlinear relationship between the sliding distance *S* and time *t* of the sample. It can be seen in Figure 6 that the specimen generally experienced an accelerated sliding stage and a decelerated sliding stage in the overall sliding process. In the initial accelerated stage, the sample acceleration declined in a short time under the forces of gravity, friction, and so on. With the extension of time, the curve changed rapidly, and the potential energy of the sample gradually converted to kinetic energy and internal energy. The kinetic energy of the sample increased first and then decreased with the passage of time. According to the law of conservation of energy, the internal energy of the sample decreased first and then increased. Therefore, the trend of displacement in the final stage of slippage gradually tended to be gentle with time, reflecting that the friction and adhesion forces between the sample and rock surfaces gradually increased.

Comparing the time-displacement curves of pure clay, fiber-reinforced clay, and PVAc-reinforced clay under different moisture contents in Figure 6, the displacement variation of the fiber-reinforced clay at the initial stage was similar to that of the pure clay sample. The effect of the fiber-reinforced clay on the bottom friction performance of the reinforced sample was mainly reflected in the deceleration stage. Sisal fiber effectively weakened acceleration by using the roughness of the surface, so that the speed of the fiber-reinforced clay decreased to lower than that of the pure clay sample in the sliding stage. The friction characteristics of PVAc-reinforced clay were reflected in both stages. In the initial acceleration stage, the PVAc formed a cementation layer on the contact surface due to the curing effect, so it had a high initial adhesion force. In this stage, the displacement of the sample increased slowly with time. With the increase of time, the slope of the curve gradually became steeper. However, due to the incomplete disappearance of the cementation layer, some potential energy was transformed into the kinetic energy of the sample displacement, and the other part was consumed by the friction energy.

Table 6 shows the acceleration–deceleration duration, average rate and residual mass of the pure clay, fiber-reinforced clay, and PVAc-reinforced clay samples. It can be seen that the sliding distance of the fiber-reinforced clay sample was smaller than that of the pure clay sample under the same sliding time, and the total sliding time of the sample increased first and then decreased with the increase in content of the sisal fiber. When the fiber content was 0.8%, the overall sliding time of the sample was prolonged, indicating that the displacement of the sample changes slowly with the increase of the sliding time under the fiber content. The sliding distance of the PVAc-reinforced clay sample was smaller than that of fiber-reinforced sample, and the sliding displacement of the sample decreased with the increase of PVAc concentration. When the PVAc concentration was 2%, the overall sliding time of the sample was prolonged, and the displacement of the sample changed slowly.

During the sliding process, the pure clay sample had a large instantaneous acceleration in the initial stage, which accelerated to peak speed in a short time. When it reached the bottom of the inclined plate, the sample failed to slow down in time and had large dynamic energy. The sliding time of the fiber-reinforced clay at the initial stage was similar to that of the pure clay sample; however, the friction between the sisal fiber and the rock interface effectively weakened the instantaneous acceleration of the sample and reduced the speed of the fiber-reinforced clay at the sliding stage. The instantaneous acceleration of PVAc-reinforced clay was small at the initial acceleration stage, and the average rates at the acceleration and deceleration stages of the sample were lower than those of fiber-reinforced clay and pure clay, indicating that PVAc effectively improved the adhesion property of the sample at the initial stage, and the high adhesion ability constrained the moving rate of the sample. In the deceleration stage, sisal fiber and PVAc improved the friction performance of the sample, so that the sample had the effect of reducing the sliding rate of the sample.

**Figure 7:**
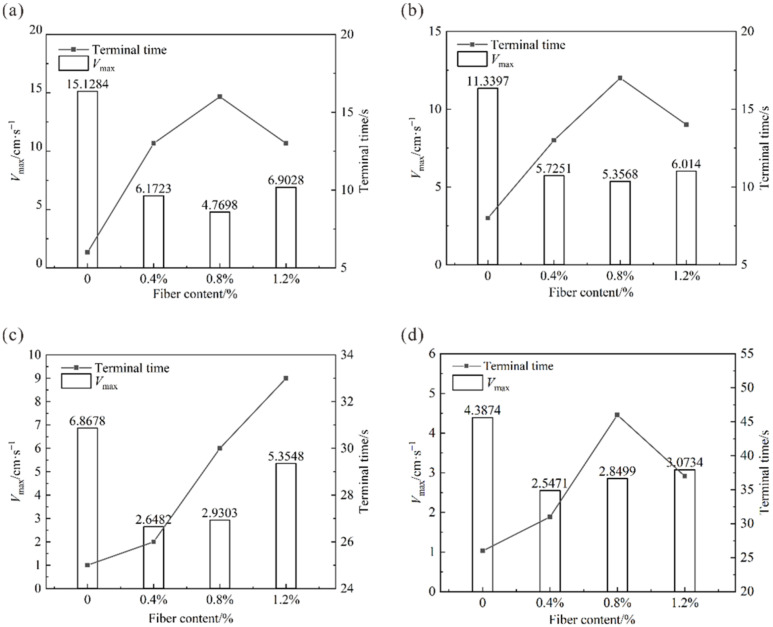
Sliding time and peak speed of fiber-reinforced clay at the moisture content of (**a**) 45%, (**b**) 40%, (**c**) 35%, and (**d**) 30%.

**Figure 8:**
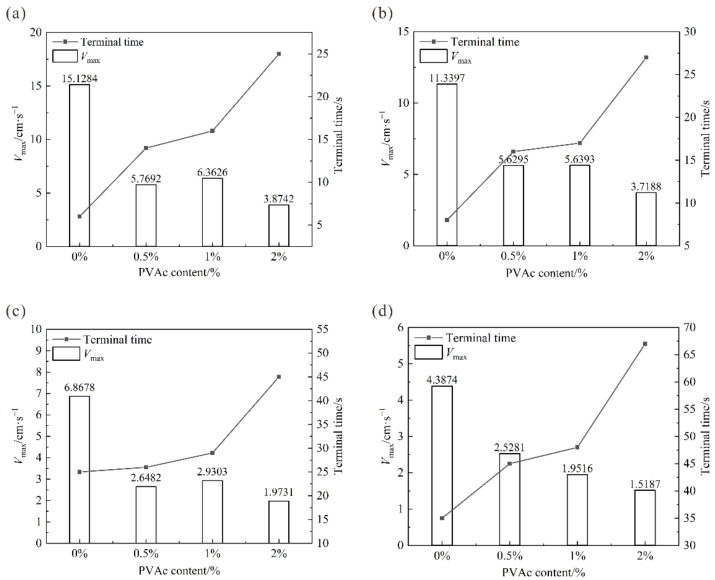
Sliding time and peak speed of PVAc-reinforced clay at the moisture content of (**a**) 45%, (**b**) 40%, (**c**) 35%, and (**d**) 30%.

In this paper, the time when the specimen slides out of the rock-like interface plate is defined as the sliding terminal time, and the maximum velocity of the specimen in the sliding process is the peak sliding velocity Vmax. The relationship curves between the peak sliding velocity and the sliding terminal time of the fiber-reinforced clay samples are shown in Figure 7. The relationship curves between the peak sliding velocity and the sliding terminal time of the PVAc reinforced clay samples are shown in Figure 8.

It can be seen from Figure 7 that the incorporation of sisal fiber reduced the peak slip velocity of the sample. When the moisture content was constant, the peak slip velocity of the sample decreased first and then increased with the increase of the sisal fiber content. When the fiber content was 0.8%, the peak slip velocity of the sample with the increase of the moisture content was 4.77 cm/s, 5.36 cm/s, 2.93 cm/s, and 2.34 cm/s, which was the minimum value under the gradient of the fiber content. When the sisal fiber was incorporated into the clay, the peak slip velocity decreased significantly by 46%, 46%, 49%, and 68% under each moisture content gradient (30–45%). When the moisture content was 30%, 40%, and 45%, the sliding end time of the sample first increased and then decreased with the increase of fiber content. When the moisture content of the sample was 35%, the sliding end time of the sample showed a continuous growth trend with the increase of fiber content. It can be seen that sisal fiber had a certain effect on improving the adhesion characteristics of clay samples, and the improvement effect was the best when the fiber content was 0.8%.

It can be seen from Figure 8 that the addition of PVAc decreased the peak slip velocity of the sample. When the moisture content was constant, the peak slip velocity of the sample decreased gradually with the increase of the concentration of PVAc. When the concentration of PVAc was 2%, the peak slip velocity of the sample with the increase of moisture content was 3.87 cm/s, 3.72 cm/s, 1.97 cm/s, and 3.07 cm/s, which was the minimum value at each concentration gradient of PVAc. When the PVAc was added to the clay, the peak sliding velocity decreased significantly. Under different moisture content gradients (30–45%), the peak sliding velocity values of the sample with 0.5% PVAc were 1.70, 2.41, 1.50, and 2.62 times lower than those of the pure clay sample. Under each moisture content gradient, the sliding end time of the sample was prolonged with the increase of the amount of PVAc. In particular, the concentration of PVAc increased from 1% to 2%, the curve of sliding terminal time increased sharply. Therefore, when the concentration of PVAc was 2%, the improvement effect was significant, which could be used as the optimal addition amount.

The bottom of the specimen was composed of countless clay particles. As the moisture content increased, free water converged at the bottom of the specimen by force of gravity, and the friction force at the interface decreased due to the lubrication of water molecules. In addition, the clay exhibited greater energy loss and softening characteristics when it was near saturation [36]. When the moisture content exceeded the liquid limit of the clay sample, the clay particles were all infiltrated by the water, so the pores were filled with free water. The free water was not bound by the electrostatic attraction of the clay particles, and it easily formed a thick water film around the soil particles. This layer of water film completely separated the soil–rock interface, resulting in a lubrication effect and reducing the friction force of the interface [37].

**Figure 9:**
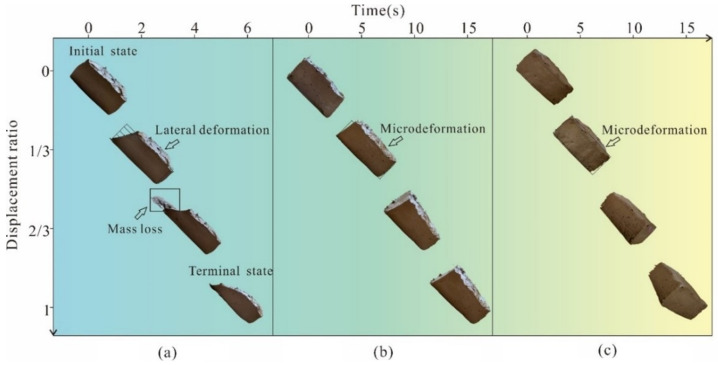
The sliding morphology of (**a**) pure clay, (**b**) fiber-reinforced clay and (**c**) PVAc reinforced clay.

It can be seen from Figure 9a that the pure clay sample was prone to significant lateral deformation when sliding for 0–2 s, and part of the clay mass adhered to the inclined plate surface when sliding for 4–6 s. When reaching the bottom of the inclined plate, the mass loss reached more than 20%. This is because when the moisture content increased, the mechanical properties of the whole sample were poor. When the clay sample moved relatively on the inclined plate surface, due to the small attraction of the clay particles in the sample, the mutual displacement between the particles changed the contact area between the bottom surface of the sample and the inclined plate, resulting in the continuous change of the molecular gravity of the whole bottom surface of the sample with the increase in sliding distance. However, some clay particles adhered to the interface of the rock mass, leading to the rearrangement of the clay microstructure on the bottom surface of the sample. This behavior could constantly adjust the contact area between the sample and the interface to a certain extent, which might have contributed to increasing the friction force [38].

Therefore, the plain clay sample was prone to deformation and cracking under the external force, and a large mass loss occurred during the sliding process. The fiber-reinforced and PVAc-reinforced methods had a good stabilizing effect on the sliding state of the sample by transmitting shear stress through the fiber network structure and strengthening the clay by PVAc chemical reaction, respectively. The mass loss was small during the sliding process, and there was no obvious lateral deformation. It shows that the fiber-reinforced clay and PVAc-reinforced clay have higher plasticity and structural stability, and can resist the sliding process (Figure 9b,c).

**Figure 10:**
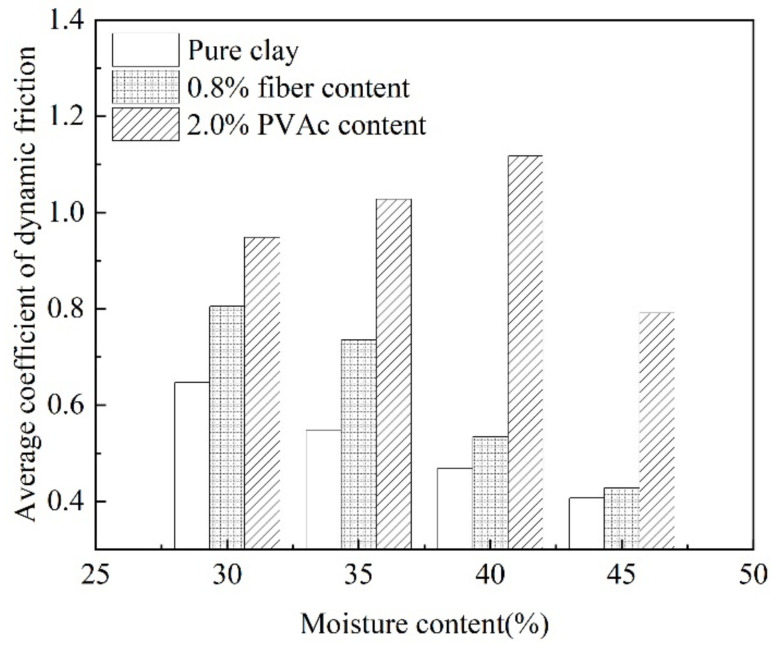
Average coefficient of dynamic friction.

Figure 9 shows that for the same distance of sliding, it took a longer time for the fiber-reinforced samples and the PVAc hardening agent-reinforced samples, which reflects that the samples reinforced by fiber materials and PVAc polymers had higher roughness and skid resistance. The dynamic friction coefficient of the sample in the sliding process reflected the change of the sliding resistance through the calculation of Equation (2). The variation range of the dynamic friction coefficient in the sliding process of the sample was small, so the average value of the dynamic friction coefficient in the sliding process of the sample was taken as the research object, as shown in Figure 10.

It can be seen from Figure 10 that the dynamic friction coefficient of the pure clay sample was generally between 0.4 and 0.65. With the increase in moisture content, the dynamic friction coefficient of the sample decreased to around 0.4 when the moisture content exceeded the liquid limit. The pure clay samples in a fluidic or semi-fluidic state, which had poor mechanical strength and stability, could not be subjected to large shear stress in the process of sliding, although there was no large shear stress in the sliding process. This resulted in a change of the contact area between the bottom of the sample and the inclined plate surface, thus reducing the overall friction characteristics and dynamic friction coefficient of the bottom of the sample. 

Different from the pure clay, the dynamic friction coefficient of fiber-reinforced clay is obviously improved in the moisture content of 30–35%. The average dynamic friction coefficient of the samples with 0.8% fiber content were 24% and 34% higher than that of the pure clay. When the moisture content was increased to the liquid limit, the average growth value of the dynamic friction coefficient was reduced to 14% and 5%, and the effect of fiber reinforcement was not obvious. Fan et al. [39] conducted an experimental study on the interfacial friction characteristics of soil under different moisture contents. The results showed that the increase of moisture content would significantly reduce the interfacial friction characteristics of soil. Under the influence of PVAc, the average dynamic friction coefficient of the sample was significantly higher than that of pure clay. Water molecules reacted with PVAc to produce enough cement. This cement had high viscosity and water retention capacity, which reduced the lubrication effect of high-moisture content on the bottom surface of the sample to a certain extent and improved the adhesion force of the bottom surface of the sample.

## 4. Mechanism

**Figure 11:**
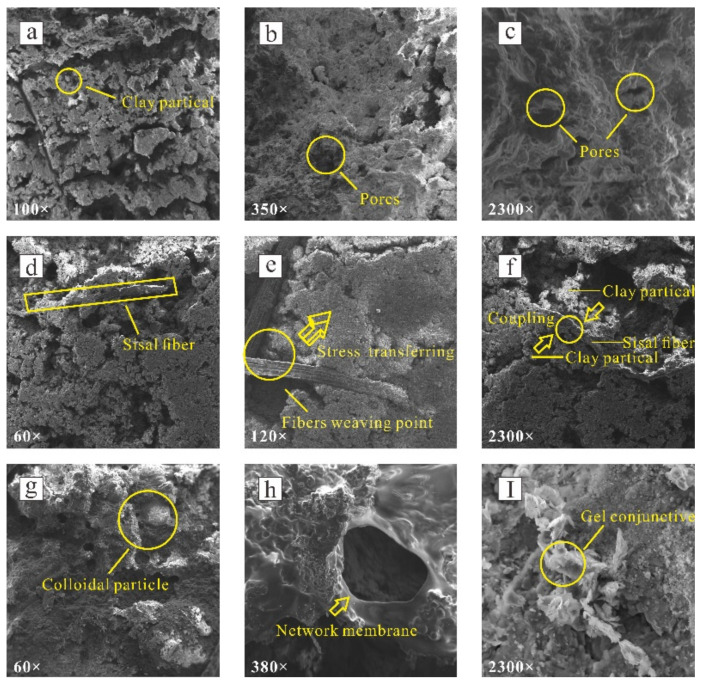
Microscopic mechanism diagram of the samples: (**a**–**c**) pure clay, magnification rates respectively 100×, 350×, and 2300×; (**d**–**f**) fiber-reinforced clay, magnification rates are 60×, 120×, and 2300×, (**g**,**h**,**I**) PVAc reinforced clay, magnification rates 60×, 380×, and 2300×, respectively.

The results of this test show that sisal fiber and PVAc possess the interfacial coupling property of soil to differing extents. The adhesion ability depends on the contact area and the roughness between the sample and the interface [40]. Resulting from the sisal fibers’ rough surface and good physical resistance ability, it effectively increased the friction characteristics between clay and rock. The friction between the fiber and the inclined plate surface was increased when the sample slid at the critical tilting angle. A single sisal fiber was ‘needle-like’ and evenly distributed in the clay at different angles to fill the pores of clay particles (Figure 11b,c). When the clay at the base-cover interface was displaced by shear stress, the cohesion and friction between clay particles and sisal fiber played a key role in that the fiber generated tensile deformation to resist part of the external load, and at the same time, the fiber transferred stress to the upper clay through the weaving point of layered accumulated fibers (Figure 11e,f). The fibers then formed an anti-restraint effect on the clay particles to prevent further deformation and enhance the strength and anti-sliding stability. 

Through the result of model sliding test, we found that fiber–clay coupling degree had great influence on the samples’ sliding conditions and states. When the moisture content was higher than the liquid limit, the coupling degree between the fiber and clay particles was poor, and the fiber had a negative impact on the critical tilting angle of the sample. The main reasons are as follows: (i) sisal fiber has weak water absorption ability. When it reaches approximate saturation or saturation state, water weakens its physical resistance ability after the fibers absorb water; (ii) in the approximate saturation state, a large amount of free water exists between the clay particles. On the one hand, free water hinders the lateral frictional resistance between the fiber and the clay particles. On the other hand, the fiber is affected by the buoyancy force of water, which reduces the reinforcement effect on the bottom surface of the sample.

**Figure 12:**
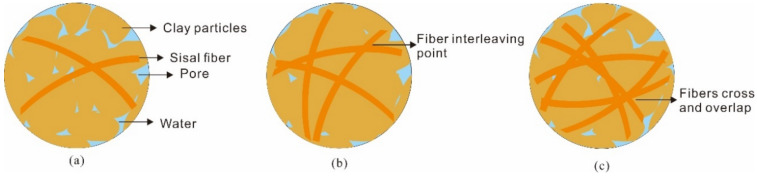
Bottom morphology of fiber-reinforced sample: (**a**) 0.4% fiber content; (**b**) 0.8% fiber content; (**c**) 1.2% fiber content.

In addition, the coupling degree of sample was affected by the fiber content. When the fiber content was 0.8%, the dynamic friction coefficient of the sample under the four moisture content conditions was generally better than that of the fiber content of 0.4% and 1.2%. When the content of sisal fiber was small, the clay sample was given priority for clay particle and clay particle contact on the microscope (Figure 12a). The static friction force of fiber-reinforced clay was not obvious. With the increase of sisal fiber content, the fiber’s pores were filled with clay particles, and the number of wrapped clay particles and the contact area of sisal fiber also increased (Figure 12b). The interfacial friction between sisal fiber and rock enhanced the ability of clay specimens to resist sliding failure and inhibit the displacement of specimens by force of gravity along the dip direction. In general, the critical angle of specimen sliding was improved. The three-dimensional network structure formed by sisal fiber also increased the anchoring effect of fiber, and mutual insertion was also more conducive to the realization of mechanical interlocking. As the content of sisal fiber continued to increase, some fibers produced repeated cross and interweaves and failed to make contact with the clay particles (Figure 12c), resulting in a failure to play the role of fiber mesh skeleton. In addition, excessive sisal fibers in some areas of the reinforced clay overlapped, cohesion and condensation split it into groups, and the uneven distribution of reinforced clay destroyed the reinforced clay structure and affected the overall reinforcement effect [41].

**Figure 13:**
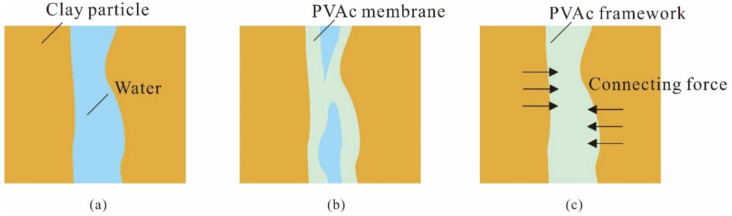
PVAc-reinforced mechanism: (**a**) pure clay sample; (**b**) 0.5% PVAc content; (**c**) 2% PVAc content.

The process of PVAc modification is shown in Figure 13a. There were many free water molecules between the clay particles in the pure clay samples, and the connection was not close. The latex particles were adsorbed by the clay sample particles after adding a small amount of PVAc. The latex particles adsorbed on the surface of different clay particles were close to each other and contacted each other, so that the clay particles connected and reduced the amount of water molecules. At this time, the elastic film formed by PVAc was more wrapped by clay particles than connected; however, this weak bonding force limited the sliding failure of the clay samples on the rock surface to a certain extent as shown in Figure 13b. With an increase of PVAc concentration, the PVAc diffused, permeated and lapped among the clay particles, forming a stable three-dimensional mesh elastic membrane structure among the clay particles, wrapping, winding, and constraining the clay particles with a wide range, filling the pores between the clay particles to play a skeletal role, making the contact between clay particles closer, as shown in Figure 13c. The presence of polymer elastic film improved the connecting force between clay particles, and this connecting force increased with an increase of PVAc concentration. When the clay particles undergo dislocation deformation under external load, it is necessary to destroy the polymer elastic film filled between them. The consolidated polymer elastic film has a certain tensile strength, which improves the strength of the clay. At the same time, PVAc can make the connection between clay–clay and clay–rock surfaces fuller and closer, so that the contact area between them increases, the force between the interface increases, and the adhesion effect of the clay–rock surface is improved.

The action mode of PVAc on clay structure mainly includes three parts: encapsulation, filling and connection. PVAc diluent forms a ‘gel conjunctive’ network membrane structure by wrapping clay particles. As the PVAc content increases, the surface tension of the ‘gel conjunctive’ is significantly enhanced, so it can effectively delay the influence of the increase of the critical tilting angle and increase the friction resistance with the inclined plate in the sliding process. The SEM images of composite samples with 2% PVAc content are shown in (Figure 11d–g). It can be clearly seen that the gel membrane structure formed by the reaction of PVAc with water effectively changed the connection form between clay particles, and the viscosity and tensile strength attached to it had a good improvement on the strength of clay.

**Figure 14:**
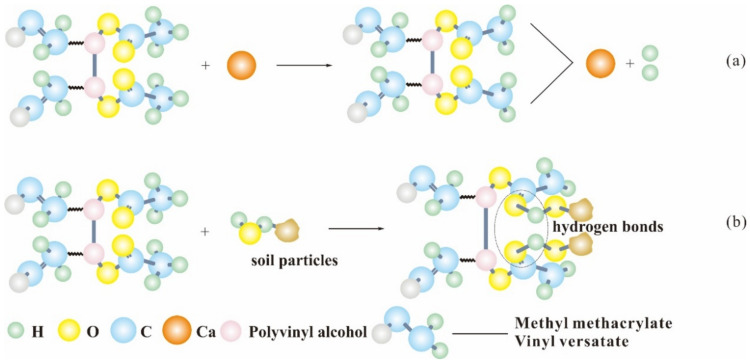
PVAc reaction mechanism; (**a**) ion substitution; (**b**) hydrogen bonding reaction.

The fiber and clay needed a good coupling degree to maximize the effect of the fiber on the friction properties of the sample bottom. Different from sisal fiber, PVAc is a kind of high-molecular polymer that has a large number of hydrophilic group polar carboxyl (–OOCCH) long chain on its surface; the latex particles in PVAc emulsion play a major role. Because of the emulsifying effect of the active agent, the surface of the latex particles had a negative charge. When the PVAc was incorporated into the clay, the latex particles were adsorbed on the surface of the clay particles due to the electrostatic attraction. With the evaporation of water, the latex particles aggregated, and its internal polymer chain gradually opened. During the mixing process of water dilution and clay, polar carboxyl (–OOCCH) long chain reacted with metal ions in clay particles by ion substitution (Figure 14a). As a result, the double electric layer on the surface of the clay particles became thin and the potential decreased. Therefore, it can increase the gravity between the clay particles and improve the strength of the clay. In addition, the hydroxyl groups on the surface of clay particles and polar carboxyl groups in the polymer formed hydrogen bonds under chemical reaction (Figure 14b). Moreover, clay particles constantly closed, aggregation with the hydrogen bonding increased, which further reinforced the clay’s stability of structure. 

## 5. Conclusions

The sliding condition of different clay properties were carried out by indoor sliding model tests. The dynamic friction coefficient of the clay sample bottom was calculated based on the curve of displacement over time recorded using video imaging and the curve fitting method. The main conclusions are as follows:

(1) Fiber-reinforced clay has good coupling characteristics with rock interfaces possessing low moisture content; it has a high critical inclination value and dynamic friction coefficient. When the moisture content was higher than the liquid limit, excessive water molecules gathered at the bottom of the sample to lubricate the sample, which weakened the effect of fiber reinforcement.

(2) The fiber content of 0.8% and PVAc content of 2% had the best effect on enhancing the sliding resistance of clay and provided good adhesion for the dangerous interfaces of rock slope within 35° and 45°, respectively.

(3) PVAc maintains a high maximum static friction force and dynamic friction coefficient under four moisture content states, and the water resistance of PVAc increases with the PVAc content.

(4) Compared with the pure clay sample, the same sliding distance of the sample after fiber- and PVAc polymer-reinforced clay needs more time. During the sliding process, the peak velocity is significantly lower than that of pure clay, and the dynamic friction coefficient is higher. When the fiber content is 0.8% or the PVAc content is 2%, the adhesion ability and anti-sliding force of clay can be improved.

(5) From a microscopic point of view, clay filled with an appropriate amount of sisal fiber forms a three-dimensional clay-fiber grid structure. PVAc was added into the clay to form a three-dimensional network elastic membrane structure through hydrogen bond and ion replacement reactions, which made the clay more fully and closely connected to the rock surface. 

(6) In future research, indoor model tests under different interface roughness, rainfall conditions, and ecological improvement methods will be further carried out, and the variation law of sliding state will be analyzed, combined with critical angle and displacement. At the same time, the shear mechanical properties of interfaces with different roughness will be the focus of future research. Combined with interface friction tests, numerical simulation will verify the feasibility of this ecological restoration method.

## Figures and Tables

**Table 1:** Basic physical properties of sisal fiber.

Cutting Length (mm)	Average Diameter (mm)	Density (g/cm^3^)	Tensile Strength (MPa)	Fracture Tensile Rate (%)	Modulus of Elasticity (GPa)
25	0.2	1.75	300.80	2.23	13.64

**Table 2:** Basic physical properties of PVAc.

Gravity	Viscosity (MPa·s)	pH	Solid Content (%)	Gel Rate (%)
1.05	630	7	43	1.50

**Table 3:** Basic physical properties of clay.

Gravity	Grain Size Analysis			Natural Moisture Content (%)	Liquid Limit (%)	Plastic Limit (%)	Plasticity Index (%)
	Clay (%)	Silt (%)	Sand (%)				
2.71	41.3	46.1	12.6	18.3	38.3	20.1	18.2

**Table 4:** Test variables of clay–rock interface.

Type of Clay Sample	Gross Mass (g)	Moisture Content (%)	Reinforced Material Content (%)
Pure clay	875	30	0
	875	35	0
	875	40	0
	875	45	0
Fiber-reinforced clay	875	30	0.4/0.8/1.2
	875	35	0.4/0.8/1.2
	875	40	0.4/0.8/1.2
	875	45	0.4/0.8/1.2
PVAc-reinforced clay	875	30	0.5/1.0/2.0
	875	35	0.5/1.0/2.0
	875	40	0.5/1.0/2.0
	875	45	0.5/1.0/2.0

**Table 5:** Clay sample descriptions of model test.

Moisture Content (%)	Fiber-Reinforced Clay				PVAc-Reinforced Clay			
	Fiber Content (%)	Average Tilting Angle (°)	Standard Deviation (°)	Maximum Static Friction (N)	PVAc Content (%)	Average Tilting Angle (°)	Standard Deviation (°)	Maximum Static Friction (N)
30	0.4	34.35	0.91	4.84	0.5	42.34	2.38	5.78
30	0.8	38.84	2.69	5.38	1	41.51	1.22	5.68
30	1.2	35.28	3.02	4.95	2	43.47	1.03	5.90
35	0.4	31.80	1.57	4.52	0.5	41.57	3.05	5.69
35	0.8	36.32	1.97	5.08	1	43.54	2.78	5.91
35	1.2	34.92	4.39	4.91	2	45.78	2.15	6.15
40	0.4	26.19	2.25	3.78	0.5	36.21	1.42	5.07
40	0.8	28.09	0.80	4.04	1	40.35	1.29	5.55
40	1.2	27.58	2.11	3.97	2	48.16	6.59	6.39
45	0.4	22.84	2.46	3.33	0.5	27.35	2.88	3.94
45	0.8	23.18	0.80	3.38	1	32.57	1.93	4.62
45	1.2	23.91	0.84	3.48	2	38.32	5.58	5.32

**Table 6:** Clay sample descriptions of model test.

Clay Properties			Acceleration Stage		Deceleration Stage		Residual Quality (g)	Mass Loss Ratio (%)
Sample Type	Material Content (%)	Moisture Content (%)	Time of Duration (s)	Average Velocity (cm/s)	Time of Duration (s)	Average Velocity (cm/s)		
Pure clay	0%	30%	1	4.39	25	2.31	847.6	3.1
	0%	35%	2	6.54	23	2.32	839.6	4.1
	0%	40%	5	8.25	3	6.95	683.3	21.9
	0%	45%	4	9.71	2	11.32	675.5	22.8
Fiber reinforced clay	0.4%	30%	2	2.53	29	1.78	863.1	1.3
	0.4%	35%	5	2.63	21	2.048	860.9	1.6
	0.4%	40%	4	5.56	9	4.74	857.2	2.0
	0.4%	45%	6	5.42	7	4.40	839.4	4.0
	0.8%	30%	1	2.85	44	1.49	865.5	1.1
	0.8%	35%	1	2.93	29	2.16	861.4	1.6
	0.8%	40%	7	4.75	10	3.34	851.5	1.5
	0.8%	45%	11	3.98	6	4.56	846.1	3.3
	1.2%	30%	1	3.07	36	1.62	865.3	1.2
	1.2%	35%	1	5.36	32	2.03	859.0	1.8
	1.2%	40%	7	4.83	8	3.61	850.4	2.8
	1.2%	45%	7	4.71	7	4.30	841.4	3.8
PVAc reinforced clay	0.5%	30%	1	2.53	44	1.38	862.4	1.4
	0.5%	35%	10	2.70	16	2.38	864.8	1.2
	0.5%	40%	10	4.18	6	3.45	860.7	1.6
	0.5%	45%	7	5.11	8	3.94	858.1	1.9
	1.0%	30%	13	1.85	35	1.05	865.7	1.1
	1.0%	35%	15	2.31	14	2.13	865.1	1.1
	1.0%	40%	9	3.54	8	3.90	862.2	1.5
	1.0%	45%	8	3.87	8	3.71	859.9	1.8
	2.0%	30%	41	1.04	26	1.30	866.1	1.0
	2.0%	35%	16	1.78	29	1.14	867.5	0.9
	2.0%	40%	15	1.99	12	2.55	865.5	0.9

## Data Availability

All the data generated by this research are included in the article.
